# Missed Opportunities for Integrated Hypertension and Diabetes Screening in Nigeria: A National Survey Analysis

**DOI:** 10.7759/cureus.107693

**Published:** 2026-04-25

**Authors:** Henry E Nwankwo, Onaedo Okeke, Ufuoma B Akporhuarho, Uzoamaka K Nduchebe, Onyinye Ngige, Princess W David, Temple I Isaac-Thomas, Mbanefo C Uyanwune

**Affiliations:** 1 Division of Allied Health, University of Chester, Chester, GBR; 2 Department of Internal Medicine, University of Nigeria, Enugu, NGA; 3 Department of Internal Medicine, Niger Delta University, Yenagoa, NGA; 4 Department of Internal Medicine, Enugu State University of Science and Technology, Enugu, NGA; 5 Department of Internal Medicine, Nnamdi Azikiwe University Teaching Hospital, Anambra, NGA; 6 Department of Internal Medicine, University of Benin Teaching Hospital, Benin, NGA; 7 Department of Community Medicine, University of Uyo, Uyo, NGA; 8 Department of Internal Medicine, City St George's, University of London, London, GBR

**Keywords:** demographic and health survey, diabetes, hypertension, integrated screening, noncommunicable diseases

## Abstract

Background: Noncommunicable diseases (NCDs) are a leading cause of morbidity and mortality globally, and the burden falls disproportionately on low- and middle-income countries. In Nigeria, hypertension and diabetes are among the most prevalent NCDs, yet diabetes screening coverage remains poorly characterised relative to blood pressure screening. This study quantifies missed opportunities for diabetes screening among adults who had already accessed blood pressure screening services and identifies the population subgroups most affected.

Methods: We conducted a secondary analysis of individual-level data from the 2023-2024 Nigeria Demographic and Health Survey (NDHS), a nationally representative survey of 39,050 women aged 15-49 years and 12,204 men aged 15-59 years. A missed opportunity was defined at the individual level as reporting a prior blood pressure measurement without a concurrent blood sugar measurement. The McNemar test compared the paired proportions of blood pressure and blood sugar screening within each sex, and multivariable logistic regression identified independent predictors of missed opportunity, adjusting for residence, education, wealth, and age.

Results: Bloodpressure screening coverage was 52.2% among women and 35.0% among men, compared with 18.6% and 19.9% for blood sugar screening, respectively. The absolute gap was 33.6 percentage points in women (McNemar p < 0.001) and 15.1 percentage points in men (McNemar p < 0.001). Among bloodpressure-screened individuals, 65.9% of women and 46.5% of men had not received blood sugar screening. In logistic regression restricted to bloodpressure-screened individuals, higher education, greater wealth, and older age were each independently associated with lower odds of missing blood sugar screening in both sexes, while residence was not a significant predictor after adjustment.

Conclusion: Among Nigerian adults who had been screened for hypertension, nearly two-thirds of women and almost half of men had never been screened for diabetes. This reflects a system-level integration failure rather than a lack of health-system contact. Embedding diabetes screening into existing blood pressure screening encounters represents a practical and scalable strategy for improving early diabetes detection in Nigeria.

## Introduction

Noncommunicable diseases (NCDs) have emerged as a major cause of morbidity and mortality globally, with low- and middle-income countries accounting for over 73% of NCD-related deaths [1]. In sub-Saharan Africa, rapid urbanization, demographic transition, and changing lifestyles have accelerated the burden of cardiovascular disease and diabetes, often in health systems historically oriented toward acute and infectious conditions [2,3].

Nigeria, Africa’s most populous country, exemplifies this epidemiological shift. Hypertension affects an estimated 28%-35% of Nigerian adults, while diabetes prevalence has increased steadily, with recent estimates ranging from 5%-7% depending on population and methodology [4-6]. Despite this burden, awareness, treatment, and control of both conditions remain low. Meta-analyses indicate that more than half of individuals with hypertension or diabetes in sub-Saharan Africa are unaware of their diagnosis [7,8].

Early detection through screening is therefore a cornerstone of NCD prevention and control. The World Health Organization’s Package of Essential Noncommunicable Disease Interventions (WHO PEN) explicitly recommends opportunistic screening for major NCD risk factors, including blood pressure and blood glucose, during routine primary healthcare encounters [9]. Integrated screening may improve efficiency in resource-constrained settings, where opportunities for repeated contact with the health system are limited.

However, evidence from multiple African settings suggests that NCD screening and care services are uneven and often under‑resourced. Reviews of diabetes care in sub‑Saharan Africa highlight systemic barriers to diagnosis and monitoring, including limited laboratory capacity, insufficient trained personnel, and inconsistent access to essential diagnostics and medications for diabetes relative to other conditions, such as hypertension [10]. Studies assessing health facility capacity for hypertension and diabetes in low-resource settings further illustrate gaps in infrastructure, protocols, and workforce readiness that may constrain consistent screening and follow-up for diabetes [11].

Research from Nigeria identifies multiple barriers to effective diabetes care, including poverty, low disease awareness, shortages of trained personnel, and systemic constraints that can limit routine diabetes assessment [12]. Evidence from primary care facility assessments indicates that, although basic screening tools such as glucometers may be available, training, guidelines, and integration into routine practice remain limited, reducing the effectiveness of diabetes screening programs [13]. These health system limitations may contribute to scenarios in which hypertension screening is more systematically implemented than comprehensive diabetes detection, despite overlapping risk profiles and shared care pathways.

This fragmentation raises an important but underexplored question: to what extent do existing hypertension screening platforms represent unrealized capacity for diabetes detection? Addressing this question requires moving beyond simple estimates of screening coverage to examine how effectively health systems use established points of contact. The 2023-2024 Nigeria Demographic and Health Survey (NDHS) provides new nationally representative data on self-reported screening for hypertension and diabetes. In this study, we use NDHS data to examine discordance between blood pressure and blood sugar screening.

This study conceptualizes blood pressure screening as evidence of health-system contact and interprets the gap between blood pressure and blood sugar screening as unrealized screening capacity for diabetes. This framing allows the assessment of not merely who is screened but how efficiently Nigeria’s health system leverages existing screening encounters. This study, therefore, aims to quantify the prevalence of missed diabetes screening opportunities among Nigerian adults who had prior blood pressure screening and identify the sociodemographic predictors of such missed opportunities.

## Materials and methods

Data source

This study is a secondary analysis of individual-level microdata from the 2023-2024 NDHS, implemented by the National Population Commission (NPC) with technical assistance from the DHS Programme. Data collection was conducted between December 2023 and May 2024 [14].

Sample design

The NDHS employed a stratified two-stage cluster sampling design to generate nationally representative estimates for Nigeria as a whole, for urban and rural areas separately, and for each of the six geopolitical zones and 36 states plus the Federal Capital Territory. In the first stage, 1,400 primary sampling units (clusters) were selected with probability proportional to size from an updated sampling frame. Stratification was achieved by separating each of the 37 subnational units into urban and rural domains, yielding 74 sampling strata. In the second stage, 30 households per cluster were selected through systematic equal-probability sampling. Data collection was successfully completed in 1,380 clusters, as 20 clusters were excluded due to security constraints during fieldwork. All women aged 15-49 years and men aged 15-59 years (in a one-third subsample of households) who were usual household members or overnight visitors were eligible for interview. The final analytical sample comprised 39,050 women (response rate 98.7%) and 12,204 men (response rate 98.2%).

The NDHS collects NCD-related data only for women aged 15-49 years and men aged 15-59 years, reflecting the standard DHS sampling framework for reproductive and adult health surveys. Although adults aged 50 years and above carry a disproportionately high burden of hypertension and diabetes, they fall outside the NDHS target age groups for NCD screening questions and were therefore unavailable for analysis.

Outcome measures

Blood pressure screening was defined as a self-reported history of ever having blood pressure measured by a doctor or other health care worker. Blood sugar screening was defined equivalently for blood sugar measurement. A missed opportunity was defined at the individual level as reporting prior blood pressure measurement without concurrent blood sugar measurement, operationalising unrealised diabetes screening capacity. Responses of "don't know" were treated as missing and excluded from analysis.

Blood pressure measurement was used as a marker of prior health-system contact because it is widely performed across all levels of the Nigerian health system, including outpatient care, antenatal services, and community outreach, and requires no consumables beyond a sphygmomanometer, making it substantially more accessible than blood glucose testing [11,13]. Blood pressure screening was not used as a proxy for clinical risk of diabetes; rather, it served as evidence that the individual had already been seen by a health worker, representing an encounter at which blood sugar testing could also have been offered.

Statistical analysis

Sociodemographic characteristics examined as explanatory variables included sex, place of residence (urban; rural), age group in five-year age bands, highest educational level attained (no education; primary; secondary; higher), and household wealth quintile (poorest to richest). These variables were selected because they represent the principal axes of socioeconomic stratification in Nigerian health research and were reported in the NDHS publication.

All analyses incorporated individual-level sampling weights to account for the complex stratified cluster design and produce nationally representative estimates. As blood pressure and blood sugar screening status are both measured on the same individuals, the two proportions are correlated (paired). The McNemar test was, therefore, used to test marginal homogeneity, that is, whether the probability of blood pressure screening differs from the probability of blood sugar screening within each sex. This is the correct test for the within-subject comparison of two paired binary proportions. McNemar p-values are reported. Multivariable logistic regression was then used to identify independent predictors of missed opportunity, with place of residence, educational level, wealth quintile, and age group entered simultaneously as covariates. Results are reported as odds ratios with 95% confidence intervals (CIs). Statistical significance was set at p < 0.05 (two-tailed) for all analyses. IBM SPSS Statistics software, version 31 (IBM Corp., Armonk, NY, USA) was used in conducting the statistical analyses.

Ethics approval

This study used publicly available, de-identified individual-level microdata from the 2023-2024 NDHS. Ethical approval for the survey was obtained by the National Health Research Ethics Committee of Nigeria and the ICF Institutional Review Board. Informed consent was obtained from all survey participants. The present secondary analysis of anonymised data did not require additional ethical approval.

## Results

Blood pressure and blood sugar screening coverage

At the national level, blood pressure screening coverage substantially exceeded blood sugar screening coverage for both women and men. Among women aged 15-49 years, 52.2% reported ever having their blood pressure measured, compared with 18.6% who reported ever having their blood sugar measured. The difference was statistically significant (McNemar p < 0.001), yielding a population-level missed opportunity gap of 33.6 percentage points. Among men, 35.0% reported blood pressure screening compared with 19.9% for blood sugar screening (McNemar p < 0.001), with a population-level missed opportunity gap of 15.1 percentage points. Among blood pressure-screened individuals, 65.9% of women and 46.5% of men had not received blood sugar screening. These findings are summarised in Table 1.

**Table 1:** Blood pressure and blood sugar screening coverage and missed opportunity prevalence, Nigeria DHS 2023–2024 Missed opportunity = blood pressure screened, blood sugar not screened. The McNemar test was used (paired proportions from the same individuals); DK = don't know responses

	Women (n = 39,050)	Men (n = 12,204)
Blood pressure screened, %	52.2	35.0
Blood sugar screened, %	18.6	19.9
Absolute difference (percentage points)	33.6	15.1
Test (McNemar)	p < 0.001	p < 0.001
Missed opportunity among blood pressure-screened individuals (weighted)	65.9%	46.5%
Valid cases (DK excluded)	38,747	11,883

Screening coverage and missed opportunities by subgroup

Table 2 presents blood pressure screening, blood sugar screening, and missed opportunity prevalence stratified by residence, education, and wealth quintile, together with the McNemar p-value for the gap between the two screening modalities within each subgroup. The gap between blood pressure and blood sugar screening was statistically significant across all subgroups (all McNemar p < 0.001). Both screening indicators increased with education and wealth and were consistently higher in urban than rural areas. Despite this, missed opportunity prevalence remained substantial across all subgroups, indicating that the integration failure is not confined to any single sociodemographic group. Among women, missed opportunity prevalence was highest among those with primary education (41.2 percentage point gap) and lowest among the richest quintile (29.3 percentage points). Among men, the gap was largest among those with primary education (17.8 percentage points) and smallest among the poorest quintile (7.3 percentage points).

**Table 2:** Blood pressure and blood sugar screening coverage, missed opportunity prevalence, and McNemar p-values by sociodemographic characteristics, NDHS 2023–2024 BP = blood pressure screened (%); BS = blood sugar screened (%); MO = missed opportunity prevalence (BP% − BS%). All p<0.001; NDHS: Nigeria Demographic and Health Survey

	Women BP%	Women BS%	Women MO%	Women p-value	Men BP%	Men BS%	Men MO%	Men p-value
Overall	52.2	18.6	33.6	<0.001	35.0	19.9	15.1	<0.001
Residence: Urban	60.6	26.6	34.0	<0.001	44.9	27.9	17.1	<0.001
Residence: Rural	44.4	11.3	33.2	<0.001	25.3	12.1	13.2	<0.001
Education: None	41.7	7.1	34.5	<0.001	14.7	5.8	9.0	<0.001
Education: Primary	57.6	16.4	41.2	<0.001	33.8	16.0	17.8	<0.001
Education: Secondary	51.7	20.3	31.4	<0.001	33.8	18.2	15.6	<0.001
Education: Higher	76.0	44.4	31.6	<0.001	60.3	41.1	19.2	<0.001
Wealth: Poorest	35.1	4.5	30.6	<0.001	10.8	3.5	7.3	<0.001
Wealth: Poorer	42.8	7.5	35.2	<0.001	20.9	8.5	12.4	<0.001
Wealth: Middle	51.5	14.3	37.2	<0.001	32.1	14.1	18.0	<0.001
Wealth: Richer	58.0	22.4	35.6	<0.001	39.8	21.9	17.9	<0.001
Wealth: Richest	68.3	39.0	29.3	<0.001	58.5	41.3	17.2	<0.001

Predictors of missed opportunity

Table 3 presents logistic regression results among individuals who had received blood pressure screening (19,907 women; 4,262 men). Among blood pressure-screened women, 65.9% had not received blood sugar screening; among blood pressure-screened men, 46.5% had not. After adjustment, a higher educational level was independently associated with lower odds of missing blood sugar screening in both women (OR 0.70, 95% CI 0.68-0.73) and men (OR 0.78, 95% CI 0.72-0.84), indicating that each additional educational level was associated with approximately 22%-30% lower odds of a missed opportunity. Similarly, the higher wealth quintile was associated with lower odds of missed opportunity in both women (OR 0.69, 95% CI 0.67-0.72) and men (OR 0.72, 95% CI 0.68-0.78). Older age group was also independently protective against missed opportunity in both sexes (women: OR 0.81, 95% CI 0.80-0.83; men: OR 0.84, 95% CI 0.82-0.87), indicating that older blood pressure-screened adults were more likely to have also received blood sugar testing. Urban residence was not a significant independent predictor in either women (OR 0.99, p = 0.773) or men (OR 0.99, p = 0.864) after adjustment.

**Table 3:** Multivariable logistic regression: predictors of missing blood sugar screening among blood pressure-screened individuals, NDHS 2023–2024 OR = odds ratio; CI = confidence interval; NDHS: Nigeria Demographic and Health Survey Sample restricted to individuals who received blood pressure screening with valid (non-don't know responses) responses for both variables.

Predictor	Women OR (95% CI)	Women p-value	Men OR (95% CI)	Men p-value
Sample (N, BP-screened)	19,907		4,262	
Urban residence	0.99 (0.92–1.07)	0.773	0.99 (0.85–1.15)	0.864
Education level	0.70 (0.68–0.73)	<0.001	0.78 (0.72–0.84)	<0.001
Wealth quintile	0.69 (0.67–0.72)	<0.001	0.72 (0.68–0.78)	<0.001
Age group	0.81 (0.80–0.83)	<0.001	0.84 (0.82–0.87)	<0.001

## Discussion

This study provides nationally representative evidence that Nigeria’s NCD screening challenge reflects inefficiencies in the use of existing screening encounters rather than a complete absence of health-system contact. Using blood pressure screening as a marker of prior exposure to a health worker, we found that a large proportion of adults who reported blood pressure screening had never been screened for diabetes. This discordance, confirmed as statistically significant in both sexes and across every sociodemographic subgroup examined (all p < 0.001), suggests systematic missed opportunities for integrated NCD screening within existing service delivery platforms.

The magnitude of this gap is particularly notable when interpreted alongside current estimates of diabetes prevalence in Nigeria. Systematic reviews and meta-analyses based on biomarker measurements have estimated adult diabetes prevalence to range from approximately 5%-7% [5,6]. In contrast, the 2023-2024 NDHS reports that only approximately 1% of respondents had been told by a health professional that they had diabetes, alongside low lifetime blood sugar screening coverage [14]. This discrepancy is consistent with underdiagnosis rather than a true difference in disease burden, a pattern previously documented across sub-Saharan Africa using population-based biomarker data [8].

From a health-systems perspective, the observed variation between blood pressure and blood sugar screening reflects missed opportunities for integration rather than lack of access. Blood pressure measurement is widely embedded within outpatient services, antenatal care, and community-based activities, whereas blood glucose testing requires additional consumables, training, and logistical support that are inconsistently available in primary healthcare settings [12,13]. In the absence of deliberate integration, diabetes screening may therefore be omitted even when contact with the health system has occurred.

The higher missed opportunity prevalence among women (65.9%) compared with men (46.5%) likely reflects the nature of the health encounters driving blood pressure screening in this population. Women of reproductive age frequently access blood pressure measurement through antenatal and postnatal care, which are not traditionally structured to include metabolic screening. This represents a particular programmatic opportunity; integrating diabetes screening into maternal health encounters would simultaneously address the missed opportunity gap and reach a population at elevated risk of gestational and postpartum diabetes.

The logistic regression findings, restricted to blood pressure-screened individuals, reveal that the missed opportunity gap falls most heavily on the least educated and poorest people within those who have already accessed care. Among blood pressure-screened women and men alike, lower educational attainment and lower wealth quintile were each independently associated with significantly higher odds of missing blood sugar screening, with each additional educational level reducing odds of a missed opportunity by approximately 22%-30%. Older age was also protective, suggesting that older adults who attend health facilities are more likely to receive comprehensive screening, possibly reflecting longer relationships with health workers or higher clinical suspicion for metabolic conditions. Urban or rural residence made no significant difference after adjustment, indicating that the socioeconomic gradient in integration quality operates independently of geography. These findings point to a structural inequity: within the population that has already overcome the barrier of accessing care, further inequalities in the quality and completeness of that care disadvantage those who are already most vulnerable.

Clinical guidance generally recommends targeted diabetes testing based on individual risk [15]; however, in settings where awareness is low and screening is not routinely implemented, diagnosis may occur late, increasing the likelihood of complications at presentation. Within this context, encounters in which blood pressure is measured represent a pragmatic platform through which diabetes screening could be more systematically incorporated, particularly in resource-constrained primary healthcare systems. These findings align with the WHO PEN, which explicitly recommends opportunistic and integrated screening for major NCD risk factors at the primary care level [9]. The present analysis extends this guidance by quantifying the gap between existing screening contacts and diabetes uptake at the population level, revealing an unrealized capacity.

These findings are consistent with patterns observed in comparable low- and middle-income country settings. In sub-Saharan Africa, pooled analyses have documented a persistently large unmet need for diabetes diagnosis, with only approximately one-third of people with diabetes having ever received a blood glucose measurement across 12 countries [8]. Also, gaps in relative facility preparedness for hypertension versus diabetes care have been documented in health facility assessments across East Africa [11], suggesting that the missed opportunity phenomenon observed in Nigeria may reflect a regional pattern of health system development rather than a country-specific failure.

Nigeria’s National Multi-Sectoral Action Plan for the Prevention and Control of NCDs emphasizes early detection and strengthening of primary healthcare systems [16]. Three specific operational priorities are indicated by these findings. First, screening protocols at all facilities where blood pressure is measured should be revised to mandate concurrent blood sugar testing, aligning with the WHO PEN framework [9]. Second, training programmes for primary healthcare workers should explicitly include integrated NCD screening competencies, with particular emphasis on antenatal care providers, given the high missed opportunity prevalence among women. Third, a reliable supply of glucometer strips and reagents must be ensured at the primary care level, as equipment availability without consumables represents a known implementation barrier in Nigerian facilities [13].

Limitations

This study has several limitations. Screening history was self-reported and subject to recall bias; the direction of this bias is uncertain, as individuals may overreport screening by confusing blood sugar testing with other blood tests or underreport if prior testing was not perceived as a formal screening encounter. The NDHS does not capture timing, frequency, or setting of screening, limiting assessment of continuity of care. Because individual risk profiles are not captured, it cannot be determined whether diabetes screening was clinically indicated at the time of each blood pressure measurement. The analysis is restricted to women aged 15-49 years and men aged 15-59 years; missed opportunities may be greater in older adults who carry the highest NCD risk, meaning the present estimates are likely conservative. Additionally, while individual-level sampling weights were applied throughout, the analyses do not fully account for the complex survey design (clustering and stratification) in the standard error estimates. Survey-adjusted methods incorporating primary sampling unit and strata identifiers would be optimal. However, given the very large sample sizes and highly significant results, the substantive conclusions are unlikely to be affected. The cross-sectional design precludes causal inference. Nonetheless, the NDHS remains the most comprehensive nationally representative source of NCD screening data in Nigeria.

## Conclusions

Nigeria has already achieved substantial population reach for hypertension screening. However, failure to integrate blood sugar testing into these encounters represents a major unrealised opportunity for early diabetes detection. Addressing this gap requires not new platforms, but better use of existing ones. Embedding routine diabetes screening within established blood pressure screening workflows at the primary healthcare level should be prioritised as a central strategy for strengthening Nigeria’s NCD response.
